# Effects of *Piper betle* Extracts against Biofilm Formation by Methicillin-Resistant *Staphylococcus pseudintermedius* Isolated from Dogs

**DOI:** 10.3390/ph16050741

**Published:** 2023-05-12

**Authors:** Arpron Leesombun, Sivapong Sungpradit, Norasuthi Bangphoomi, Orathai Thongjuy, Jantraporn Wechusdorn, Sunee Riengvirodkij, Jakaphan Wannawong, Sookruetai Boonmasawai

**Affiliations:** 1 Department of Pre-Clinic and Applied Animal Science, Faculty of Veterinary Science, Mahidol University, Nakhon Pathom 73170, Thailand; arpron.lee@mahidol.edu (A.L.); sivapong.sun@mahidol.edu (S.S.); norasuthi.ban@mahidol.edu (N.B.);; 2 Prasu-Arthorn Animal Hospital, Faculty of Veterinary Science, Mahidol University, Nakhon Pathom 73170, Thailand

**Keywords:** ethanolic extracts, *Piper betle*, *Piper sarmentosum*, *Piper nigrum*, pyoderma, biofilm, hydroxychavicol

## Abstract

Emergence of methicillin-resistant *Staphylococcus pseudintermedius* (MRSP) isolated from dogs with cutaneous and wound infections has significantly impacted veterinary medicine. This study aimed to isolate *S. pseudintermedius* from canine pyoderma and investigate the effects of ethanolic extracts of *Piper betle* (PB), *P. sarmentosum* (PS), and *P. nigrum* (PN) on the bacterial growth and biofilm formation of *S. pseudintermedius* and MRSP. Of the isolated 152 isolates, 53 were identified as *S. pseudintermedius* using polymerase chain reaction, and 10 isolates (6.58%) were identified as MRSP based on the presence of *mecA*. Based on phenotype, 90% of MRSPs were multidrug-resistant. All MRSP had moderate (10%, 1/10) and strong (90%, 9/10) biofilm production ability. PB extracts were the most effective in inhibiting planktonic cells, and the minimum inhibitory concentration at which ≥50% of the isolates were inhibited (MIC_50_) was 256 µg/mL (256–1024 µg/mL) for *S. pseudintermedius* isolates and 512 µg/mL (256–1024 µg/mL) for MRSP isolates. The MIC_90_ for *S. pseudintermedius* and MRSP was 512 µg/mL. In XTT assay, PB at 4× MIC showed an inhibition rate of 39.66–68.90% and 45.58–59.13% for *S. pseudintermedius* and MRSP, respectively, in inhibiting biofilm formation. For PB at 8× MIC, the inhibition rates for *S. pseudintermedius* and MRSP were 50.74–81.66% and 59.57–78.33%, respectively. Further, 18 compounds were identified in PB using gas chromatography–mass spectrometry, and hydroxychavicol (36.02%) was the major constituent. These results indicated that PB could inhibit bacteria growth of and biofilm formation by *S. pseudintermedius* and MRSP isolated from canine pyoderma in a concentration-dependent manner. Therefore, PB is a potential candidate for the treatment of MRSP infection and biofilm formation in veterinary medicine.

## 1. Introduction

*Staphylococcus pseudintermedius* is a Gram-positive and coagulase-positive bacteria belonging to the *Staphylococcus intermedius* group. They mainly colonize on the skin and mucous membranes of many wildlife and companion animals; therefore, this bacterium is considered a public health concern because of its zoonotic potential [1,2]. *Staphylococcus pseudintermedius* is an opportunistic bacterium that can infect dogs and is the predominant pathogen in pyoderma, otitis externa, and systemic infections in the urinary, respiratory, and reproductive tracts [3]. These conditions frequently require the administration of systemic antimicrobial agents for the treatment [4]. Over the past decade, methicillin-resistant *S. pseudintermedius* (MRSP), a strain carrying the *mecA* gene, has been increasingly isolated from both healthy and infected dogs [5]. *mecA* is encoded in the staphylococcal chromosomal cassette mec (SCCmec), a motile genetic element that plays a major role in the antibiotic resistance of *Staphylococcus* spp. [6]. *mecA* encodes the production of a modified penicillin-binding protein that results in low affinity for several β-lactams; hence, these antimicrobials do not affect bacterial cell wall construction [7,8,9]. MRSP often exhibits resistance to certain classes of antimicrobials, such as penicillins, tetracyclines, and macrolides; moreover, the emergence of multidrug resistance poses a challenge for the treatment of both animal and human infections [5,8,10]. Potential risk factors for MRSP infection include frequent antimicrobial use to treat chronic or intermittent infections like pyoderma or surgical site infections, as well as previous exposure to MRSP in a hospital or within the family [11]. Some studies have reported that the development of biofilm by pathogens is related to their resistance to antimicrobial agents, potentially contributing to chronic infections [12,13].

Biofilms are a consortium of microorganisms that stick to each other because of the complex assembly of multiple bacterial cellular matrices. During biofilm formation, bacterial cells first attach to a surface, multiply further, and accumulate at the primary adhesion site to form microcolonies. The bacteria in these microcolonies produce extracellular matrix, which is a defining characteristic of biofilm formation. This matrix is made up of polysaccharides, proteins, and extracellular DNA. Thereafter, the biofilm matures into three-dimensional structures and subsequently undergoes a disassembly process, leading to the dissemination of the bacterial cells [14,15,16]. Most *S. pseudintermedius* and MRSP can form biofilms, and the majority of isolates are classified as strong or moderate biofilm producers [17]. Biofilms can protect the bacteria themselves from host defense, disinfectants, and antibiotics. They block or retard the penetration of certain antibiotic molecules. Thus, a high-level of antibiotics (up to 1000 times) is required [18]. Moreover, biofilm formations can cause chronic infections and the emergence of antibiotic-resistant [19,20]. This may be a significant virulence factor contributing to the rapid spread of this bacterium in veterinary hospitals around the world [17]. In veterinary medicine, the emergence of MRSP is a new challenge because of the limited therapeutic options [9]. Thus, new compounds or natural extracts as alternative treatments for bacterial resistance are required, including essential oils and plant extracts that are popular as natural remedies in human and veterinary medicine [21].

Plant extracts containing several phytochemicals exhibit significant antimicrobial properties against various pathogens, including microbial resistant strains [22,23]. Most of the active extracts contain tannins, (poly) phenols (including flavonoids, lignans, and coumarins), terpenoids, or alkaloids; these have been previously reported as active compounds against methicillin-resistant *Staphylococus aureus* (MRSA) [23].

Piperaceae plants, which comprise approximately 1000 species, are commonly found in the tropical regions of India, Southeast Asia, and Africa [24]. In Thailand, 40 species of this plant have been identified [25]. Pharmacologically, Piperaceae plants exhibit antibacterial, antioxidant, gastrointestinal protective, anticancer, insecticidal, and antiprotozoal properties [26,27,28]. Piper plants, such as *Piper betle* (PB), *P. sarmentosum* (PS), and *P. nigrum* (PN), exert antibacterial effects against Gram-negative bacteria including *Escherichia coli* and *Pseudomonas aeruginosa*, Gram-positive bacteria including *S. aureus*, and fungi including *Candida albicans*. These plants have also been reported to exert effects against multidrug-resistant (MDR) bacteria in humans, such as metallo-β-lactamase (MβL)-producing *P. aeruginosa* and MβL-producing *Acinetobacter baumannii* [29,30,31,32,33]. The minimum inhibitory concentration (MIC) of PB ethanolic extracts against *E. coli* and *P. aeruginosa* was 0.03–0.4% *w*/*v*, whereas that against MRSA was 0.0078–0.0156% *w*/*v* [31]. Additionally, the ethanolic extracts of PB leaves exhibited strong antibacterial activity against clinical isolates of avian pathogenic *E. coli* with MIC and minimum bactericidal concentration (MBC) values of 0.5–1.0 mg/mL [34].

Although the antibacterial effects of piper plants on some pathogenic bacteria have been studied, knowledge on the effects of Piperaceae on *S. pseudintermedius* is limited. In this study, we evaluated the effect of crude ethanolic extracts of PB, PS, and PN on planktonic cells and biofilm formation by *S. pseudintermedius* and MRSP isolated from canine clinical samples.

## 2. Results

### 2.1. Plants Extraction Yield

The weights of the final crude extracts of PB, PN, and PS were 30.3, 25.6, and 16.6 g, respectively, and the yields (%) of the extracts based on their dry weights were 3.56%, 2.56%, and 3.77%, respectively.

### 2.2. Bacterial Identification

Of the 152 clinical canine isolates, 73 (48.03%) were coagulase-positive *Staphylococcus* spp., and 53 (34.87%) of these were identified as *S. pseudintermedius* through PCR (Figures S1 and S2). Ten of the *S. pseudintermedius* isolates were *mecA*-positive (6.58%), whereas the remaining (51.97%, 79/152) were unidentified Gram-negative bacteria. The phylogenetic tree of nucleotide sequences from a 701 base pair (bp) fragment of 16S rRNA and 254 bp fragment of *mecA* in *S. pseudintermedius* was shown in Figures S3 and S4.

### 2.3. Biofilm Classification and Antimicrobial Susceptibility Characteristics 

**Figure 1:**
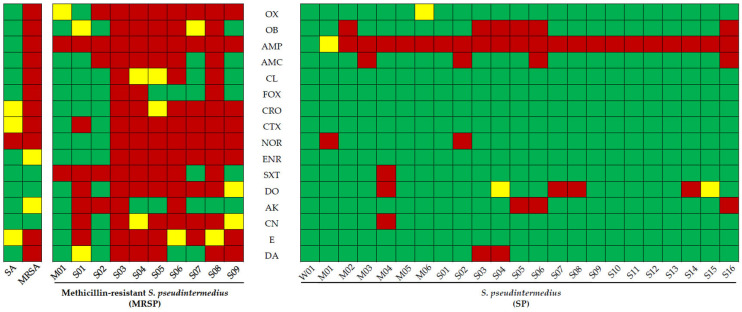
Antimicrobial susceptibility profiles of *Staphylococcus aureus*, methicillin-resistant *S. aureus*, methicillin-resistant *Staphylococcus pseudintermedius*, and *S. pseudintermedius* were determined using the disk-diffusion method. Red squares indicate resistance; green squares indicate susceptibility, and yellow squares indicate intermediate characteristics. Abbreviations: SA, *Staphylococcus aureus* ATCC 25923; MRSA, methicillin-resistant *S. aureus* ATCC 33591; W, weak biofilm producer; M, moderate biofilm producer; S, strong biofilm producer; OX, oxacillin (1 µg); OB, cloxacillin; AMP, ampicillin (10 µg); AMC, amoxicillin–clavulanic acid (30 µg); CL, cephalexin (30 µg); FOX, cefoxitin (30 µg); CRO, ceftriaxone (30 µg); CTX, cefotaxime (30 µg); NOR, norfloxacin (10 µg); ENR, enrofloxacin (5 µg); SXT, sulfamethoxazole/trimethoprim (25 µg); DO, doxycycline (30 µg); AK, amikacin (30 µg); CN, gentamicin (10 µg); E, erythromycin (15 µg); DA, clindamycin (2 µg).

The biofilm-forming ability of the bacteria was evaluated via crystal violet assays (Figures S5 and S6). In this study, all *S. pseudintermedius* and MRSP isolates were identified as biofilm producers. *S. aureus* ATCC 25923 and MRSA ATCC 33591 were classified as strong biofilm producers. One MRSP isolate was considered a moderate biofilm producer; almost all MRSP were considered strong biofilm producers. In contrast, 1 isolate of *S. pseudintermedius* was identified as a weak biofilm producer; 6 were moderate biofilm producers; 16 were strong biofilm producers (Figure 1 and Figure S6).

In this study, 90% of MRSPs, which are moderate and strong biofilm producers, were multidrug-resistant. The 1 isolate (4.35%) of *S. pseudintermedius*, which is a weak biofilm producer, was susceptible to all antimicrobials. Another 10 isolates were resistant to only one antimicrobial; 7 were resistant to two antimicrobial classes; 3 were resistant to three antimicrobial classes, and 1 was resistant to four antimicrobial classes (Figure 1 and Table S1).

### 2.4. Antibacterial Effects on Planktonic Cells

The MIC values of three ethanolic extracts of PB, PN, and PS are presented in Table 1. PB had the lowest MIC_50_ against *S. pseudintermedius* and MRSP than the others. The MIC range (256–1024 µg/mL) of PB against *S. pseudintermedius* and MRSP isolates was equal. PN and PS exhibited higher potency to inhibit *S. pseudintermedius* than MRSP due to their lower MIC_50_ values and MIC range (Table 1). PB exerted bactericidal effects against *S. pseudintermedius* and MRSP (MBC/MIC ratio ≤ 4). PN showed a bactericidal effect on *S. pseudintermedius* isolates (69.5%), whereas PS showed a bactericidal effect on 90% of *S. pseudintermedius* and all MRSP isolates.

### 2.5. Antibiofilm Effects against S. pseudintermedius and MRSP

**Figure 2:**
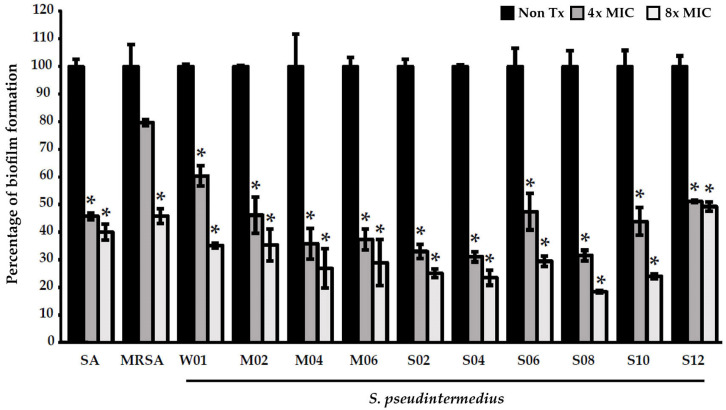
Effects of *Piper betle* ethanolic extracts on the biofilm formation of *Staphylococcus aureus*, methicillin-resistant *S. aureus*, and *Staphylococcus pseudintermedius* after 24 h of treatment. Each bar shows the mean ± SD of three experiments per group. * indicates that the differences between the control and treatments were statistically significant (*p* < 0.05). Abbreviations: SA, *Staphylococcus aureus* ATCC 25923; MRSA, methicillin-resistant *S. aureus* ATCC 33591; W, weak; M, moderate; and S, strong biofilm producers.

**Figure 3:**
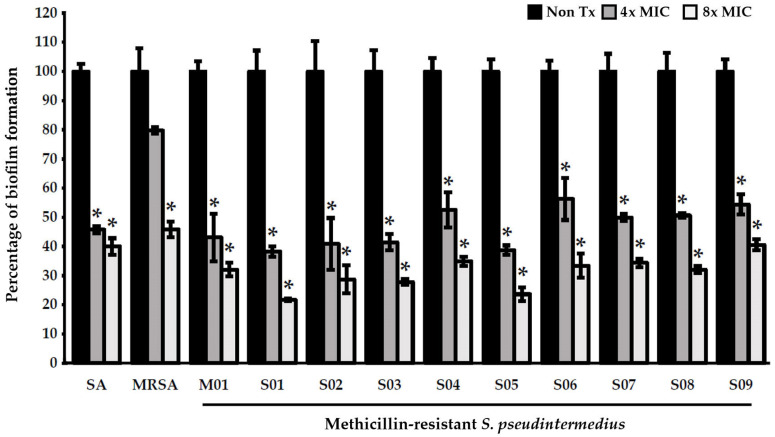
Effects of *Piper betle* ethanolic extracts on the biofilm formation of *Staphylococcus aureus*, methicillin-resistant *S. aureus*, and methicillin-resistant *Staphylococcus pseudintermedius* after 24 h of treatment. Each bar shows the mean ± SD of three experiments per group. * indicates that the differences between the control and treatment groups were statistically significant (*p* < 0.05). Abbreviations: SA, *Staphylococcus aureus* ATCC 25923; MRSA, methicillin-resistant *S. aureus* ATCC 33591; W, weak; M, moderate; and S, strong biofilm producers.

Among the extracts, only PB effectively exerted antibiofilm effects against *S. pseudintermedius* and MRSP at 4× MIC (2048 µg/mL) and 8× MIC (4096 µg/mL) (*p* < 0.05) (Figure 2 and Figure 3 and Table S2). PB increased the antibiofilm effects in a concentration-dependent manner. At 8× MIC (4096 µg/mL), PB demonstrated a significant activity against MRSA.

### 2.6. Chemical Composition of PB Extracts

**Figure 4:**
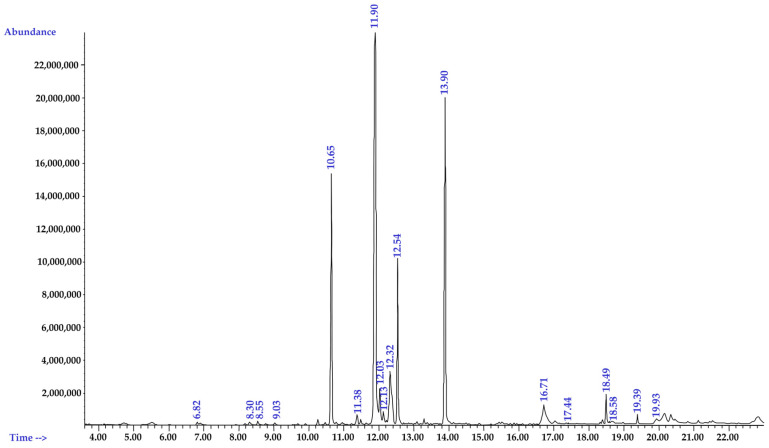
Chromatogram of the main components of *Piper betle* ethanolic extracts determined through gas chromatography–mass spectrometry.

Because PB was the most effective in inhibiting bacterial growth and biofilm formation, the components of the extracts were further investigated by gas chromatography–mass spectrometry (GC–MS). A total of 18 compounds were identified, accounting for 88.97% of the PB extracts (Table 2). The major components were hydroxychavicol (36.02%), allylpyrocatechol diacetate (17.56%), and chavibetol (12.3%). The chromatogram of the main components of PB is presented in Figure 4.

## 3. Discussion

To the best of our knowledge, this is the first study to report the antimicrobial and biofilm-inhibitory effects of PB ethanolic extracts on clinically isolated *S. pseudintermedius* and MRSP from dogs. Canine pyoderma is a highly prevalent bacterial skin infection in dogs, and *S. pseudintermedius* accounts for up to 92% of the pathogens isolated from companion dogs [3]. Studies have investigated the occurrence of MRSP colonization and contamination across diverse dog populations in different countries, with rates of up to 59% [3,35,36]. Nakaminami et al. reported a high prevalence of *S. pseudintermedius* and MRSP in Japan, with prevalence rates of 74.5% (82/110) and 34.1% (28/82) for *S. pseudintermedius* and MRSP collected from pyoderma lesions, respectively [37]. The prevalence rate of MRSP identified based on *mecA* was 6.58% (10/152); Rana et al. reported prevalence rates of 45.3% and 6% for *S. pseudintermedius* and MRSP in dogs in Bangladesh, respectively. All isolated bacteria were resistant to more than three antimicrobial classes [35]. MRSP prevalence in *S. pseudintermedius* isolated in this study was 1.5 times lower than that previously reported by Jantorn et al. in Thailand in 2021 (18.87%, 10/53 vs. 28.30%, 15/53) [38]. The difference in *S. pseudintermedius* prevalence depends on the sample size, collection sites, and geographic region [9,35,39].

Oxacillin susceptibility test using the disk-diffusion method is recommended for basic screening of methicillin resistance in staphylococcus [9]. Contrary to *S. aureus*, cefoxitin disk diffusion method is inappropriate to screen for methicillin resistance and *mecA* expression in *S. pseudintermedius* [40]. When staphylococci showed a methicillin-susceptible phenotype by the oxacillin susceptibility test, PCR targeting *mecA* is a more reliable method for confirmation of methicillin-resistant [38,40]. In this study, 10 isolates were *mecA*-positive, and 8 of these had the oxacillin-resistant phenotype. Two *mecA*-positive isolates were susceptible to oxacillin and oxacillin-intermediate each. Previous observations have indicated a disparity between the detection of *mecA* and the lack of associated oxacillin resistance in *S. pseudintermedius* and *S. aureus* [41,42,43,44]. To prevent false-positive or false-negative outcomes, a combination of genotypic and phenotypic testing is essential [45]. This is an area of concern for the treatment of oxacillin-susceptible, *mecA*-positive *S. pseudintermedius* infection because once the bacteria are exposed to β-lactam antibiotics, the risk of treatment failure increases [46].

This study confirmed the trend of infection by multidrug-resistant bacteria in canines. From 23 isolates of *S. pseudintermedius*, only 1 (4.35%) was susceptible to all antimicrobials, whereas 4 (17.39%) showed resistance to ≥3 classes, exhibiting multidrug resistance. All 10 MRSP isolates in this study were highly resistant to ampicillin; 9 (90%) exhibited multidrug resistance, and 2 (20%) were resistant to nine antimicrobial classes. MRSP is often resistant to commonly used β-lactam antibiotics and antimicrobial drugs. Systemic antimicrobial agents approved for veterinary use may not be effective or viable for treating MRSP infections [39]. MRSP isolates are reportedly resistant to β-lactams and other antibiotics, including chloramphenicol (53.33%), trimethoprim (73.33%), clindamycin (73.33%), clarithromycin (80.00%), ciprofloxacin (93.33%), and tetracycline (100%) [38]. Furthermore, the presence of *mecA* significantly affects the antibiotic resistance of *S. pseudintermedius* [47]. Our findings are consistent with those of other studies [38,48]. MRSP isolates exhibited multidrug resistance, all isolates of the MRSPs being resistant to ampicillin; 80% resistant to oxacillin, cefotaxime, and sulfamethoxazole/trimethoprim; 70% resistant to norfloxacin, enrofloxacin, and doxycycline; and 60% resistant to amoxicil-lin–clavulanic acid, ceftriaxone, gentamicin, and erythromycin. The antimicrobial drugs used as the first-line treatment for dogs with dermatological problems, including bacterial infection, open wounds, and allergy and skin mass, included amoxicillin–clavulanic acid (52.3%), enrofloxacin (27.6%), and marbofloxacin (7.2%) administered through the parenteral route and cephalexin (38.4%), amoxicillin–clavulanic acid (22.3%), and enrofloxacin (12.7%) administered through the oral route [49]. These MRSP isolates had multidrug resistance profiles that are frequently resistant to the first-line antimicrobials prescribed for dogs with dermatological problems, leading to difficulties in managing MRSP infection.

Increased biofilm production contributes to the failure of *S. pseudintermedius* treatment, which is a growing concern in veterinary medicine. Biofilm formation may also be an important virulence factor that allows *S. pseudintermedius* colonization in dogs. These factors facilitated the survival of bacteria in the upper respiratory tract [17]. Strong biofilm producers induced more inflammatory reactions than weak biofilm producers [50]. Biofilm-producing bacteria were characterized as weak, moderate, and strong biofilm producers based on crystal violet staining, a reliable method to quantify the biofilm biomass and the matrix of both living and dead cells [51]. After treatment with antimicrobials, the XTT assay was employed to assess the metabolic activity of biofilm cells [52]. In this study, the *S. pseudintermedius* isolates included weak (4.35%, 1/23), moderate (26.09%, 6/23), and strong (69.57%, 16/23) biofilm producers, whereas the MRSP isolates included moderate (10%, 1/10) and strong (90%, 9/10) biofilm producers. Jantorn et al. reported similar results that most *S. pseudintermedius* isolates were moderate and strong biofilm producers. Specifically, they identified 26 (49.05%) as moderate biofilm producers and 22 (41.50%) as strong biofilm producers. Contrarily, only five (9.43%) belonged to weak biofilm producers [38]. Another study by Singh et al. revealed that 136 of the 140 (96%) *S. pseudintermedius* isolates were moderate to strong biofilm producers. Both *S. pseudintermedius* and MRSP being able to produce biofilm, the biofilm producing characteristics had no difference (*p* = 0.8) [17]. Silva et al. reported a positive correlation between biofilm formation and multidrug resistance. In comparison to non-MDR isolates, MDR strains produced a notably greater quantity of biofilm materials [10]. However, antibiotic resistance and biofilm production ability were not significantly correlated [38]. Here, MRSP isolates that exhibited multidrug resistance profiles were strong biofilm producers; however, additional samples are needed to confirm the correlation between biofilm formation and multidrug resistance.

Several studies have demonstrated that PB exerts an antimicrobial effect on various pathogens, including Gram-positive and Gram-negative bacteria and yeasts [31,34,53,54]. In addition, it reportedly inhibits bacterial biofilm formation [31]. Of the extracts of all Piperaceae species, including PB, PN, and PS, that of PB had the most significant bactericidal effect on the *S. pseudintermedius* and MRSP isolates, with MIC values of 256–1024 µg/mL. PB extracts also exerted inhibitory effects against bacterial biofilm formation. The biofilm inhibition capacity of the extracts was considered “too active” when biofilm inhibition was >50% [55]. PB extracts were highly effective in inhibiting the biofilm formation of both *S. pseudintermedius* (n = 10) and MRSP (n = 10) isolates from canine skin infection sites. PB extracts at 4× MIC (2048 µg/mL) and 8× MIC (4096 µg/mL) could inhibit biofilm formation by >50% in a concentration-dependent manner. Kulnanan et al. reported that the ethanolic extract of PB leaves at 1/8×, 1/4×, and 1/2× MIC significantly inhibited the biofilm formation of avian pathogenic *E. coli* (90%) [34]. Antibiofilm molecules interfere with quorum-sensing pathways and adhesion mechanisms and disrupt extracellular DNAs, proteins, lipopolysaccharides, exopolysaccharides, and secondary messengers involved in various signaling pathways [56]. The antibiofilm mechanisms of PB should be investigated in future studies.

Hydroxychavicol is a major phytochemical compound found in PB ethanolic extracts. The results of this study are consistent with those of the study by Kulnanan et al. [34], who reported that PB leaf extracts are majorly composed of hydroxychavicol (54.61%). Hydroxychavicol is an allylbenzene class of natural product; it exhibits antimutagenic, anticarcinogenic, antioxidant, anti-inflammatory, xanthine oxidase inhibitory, and antimicrobial properties [57,58,59]. It also exhibits an inhibitory effect on fungal species, such as *Aspergillus*, *C. albicans*, and *C. glabrata*. Furthermore, it inhibits the growth of biofilms produced by *C. albicans* and reduces preformed biofilms, probably via membrane disruption [60]. Hydroxychavicol affects Gram-positive and Gram-negative bacteria, which mediate bacterial cell death via reactive oxygen species generation and DNA damage [59]. In response to DNA damage that leads to cell cycle arrest, hydroxychavicol has been found to suppress the expression of SulA, a protein regulated by the SOS response, which triggers DNA repair and mutagenesis [59]. In addition, hydroxychavicol or allylpyrocatechols reportedly cause cell wall disruption in *Streptococcus sanguinis* by blocking UDP-N-acetylglucosamine enolpyruvyl transferase, which is an important step in the peptidoglycan biosynthetic pathway [31]. However, studies on the antibacterial activity of allylpyrocatechol diacetate and chavibetol are limited. Allylpyrocatechol diacetate and other propenylphenols, chavicol, chavibetol, allylpyrocatechol, and chavibetol acetate in PB leaf extracts exhibited a favorable response to fungicidal and nematocidal activities [61].

Hydroxychavicol is likely responsible for the observed antibacterial and antibiofilm effects. However, the study requires further investigation in a larger population to confirm the results. Additionally, the compounds found in PB should be further investigated as PB is a promising treatment for MRSP infection and biofilm formation. This is a potentially high-impact line of research as MDR bacteria are responsible for most cases of dermatitis encountered in veterinary medicine.

## 4. Materials and Methods

### 4.1. Plant Material and Extraction

**Figure 5:**
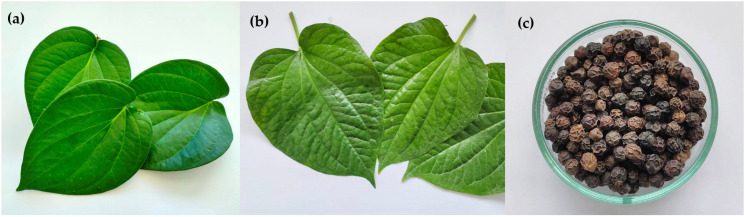
Leaves of *Piper betle* L. (**a**) and *P. sarmentosum* Roxb. (**b**) and dried seeds of *P. nigrum* L. (**c**).

Leaves of *P. betle* L. and *P. sarmentosum* Roxb. and dried seeds of *P. nigrum* L. were collected from central Thailand from April to May 2020 (Figure 5). All plants were identified and stored at the Sireeruckhachati Nature Learning Park, Faculty of Pharmacy, Mahidol University. The plant serial numbers were as follows: *P. betle* L., PBM–005510–1; *P. nigrum* L., PBM–005504–6; and *P. sarmentosum* Roxb., PBM–005491–2. The leaves of PB (5000 g) and PS (6000 g) were washed with tap water and oven-dried at 60–70 °C for 48–72 h. The seeds of *P. nigrum* (1000 g) were dried and ground to small pieces. The test plants were extracted by treating with 97% ethanol (Sigma-Aldrich, St. Louis, MO, USA) at room temperature (RT) for a period of 3 days, filtered through sterile gauze, and evaporated to dryness under reduced pressure at 40 °C using a rotary evaporator (Rotavapor R-200/205, BÜCHI, Flawil, Switzerland). The extracts were then lyophilized using a Freeze Dry Vacuum System (Labconco, Kansas City, MO, USA). The final crude extracts were weighed, and the yields of the extracts were calculated based on their dry weights. The crude extracts were dissolved in dimethyl sulfoxide (DMSO) at 100 mg/mL before use.

### 4.2. Bacterial Isolation and Identification

This study was conducted in strict accordance with the recommendations in animal care. The protocol was approved by the Faculty of Veterinary Science-Animal Care and Use Committee (MUVS-2019-02-08) and the Faculty of Veterinary Science-Institutional Biosafety Committee (IBC/MUVS-B-009-2563). Skin swabs were collected from dogs presenting to the Veterinary Medical Teaching Hospital, Prasu-Arthorn Animal Hospital, Thailand during the year 2020, with clinical signs of pyoderma, characterized by scaly and itchy skin, skin redness, and lesions that frequently develop pustules and ulcers [62]. Skin swabs samples were collected from 147 dogs not treated with any antimicrobials within 1 month. Then, each sample was streaked on 5% sheep blood agar (Clinical Diagnostics Ltd., Bangkok, Thailand) and incubated overnight at 37 °C. Suspected colonies were subjected to Gram’s staining, and the positive ones showing the characteristics of *Staphylococcus* were further streaked onto Mannitol salt agar plates (Clinical Diagnostics Ltd., Bangkok, Thailand) and incubated at 37 °C for 24 h [63]. The presumptive colonies of staphylococci on Mannitol salt agar were further tested via catalase and coagulase production. All coagulase-positive isolates were further examined using molecular methods, with specific primers for *S. pseudintermedius* and *mecA* as previously described [64,65,66,67].

### 4.3. DNA Extraction for Staphylococcal Species

The genomic DNA of *Staphylococcus* spp. isolates was extracted using the boiling method with some modifications [68]. Briefly, a single colony was picked and boiled in 100 µL of RNase-free water in a 95 °C block heater for 10 min and then centrifuged at 1300 rpm for 10 min to remove cell debris. The supernatant was transferred to a new tube, and its concentration and purity were measured using a Nanodrop One spectrophotometer at 260/280 and 260/230 ratios, respectively, and stored at −20 °C until further use.

### 4.4. Detection of mecA Encoding in S. pseudintermedius Isolates through Single PCR

The DNA extracted by boiling method was used as a template for PCR to amplify 310 bp of *mecA* [69] and 926 bp of *nuc* [64]. The single PCR reaction mixtures were composed of the following: 12.5 µL of Taq polymerase TopTaq Master Mix and 0.5 µL of each of the forward and reverse primers (10 µm), 2.5 µL of CoralLoad Concentrate (Qiagen), 2 µL of DNA template (50 ng/µL), and up to 25 µL of molecular biology grade water. MRSA (ATCC 33591) was used to verify the specificity of *nuc* primers. Amplifications for *mecA* and 16S rRNA were performed in a T100TM thermal cycler. The thermocycling conditions were shown in Table 3. Amplicons were electrophoresed on 2% Agarose gels, stained with GelRed^TM^ (Biotium, Hayward, CA, USA), and visualized using a c300 UV transilluminator (Azure Biosystems, Dublin, CA, USA). All primers for PCR are presented in Table 4.

All *S. pseudintermedius* 16S rRNA and *mecA* sequences were compared with sequences available in the GenBank database using The Basic Local Alignment Search Tool. Multiple alignments of all nucleotide sequences were conducted using the ClustalW web-based tool (https://www.genome.jp/tools-bin/clustalw, accessed on 19 April 2023) [71]. Phylogenetic trees were reconstructed using maximum likelihood analysis with bootstrapping (100 replications) in the advanced mode of the phylogeny.fr web server (http://www.phylogene.fr/, accessed on 19 April 2023) [72].

Published 16S rRNA sequences in the GenBank database originating from other global locations were used to compare all sequences including *S. aureus* (MZ603719), *Staphylococcus canis* (NR_181183), *Staphylococcus felis* (MN148648), *Staphylococcus schleiferi* (KX242542), and *Staphylococcus haemolyticus* (EF692529) for the 16S rRNA phylogenetic tree. The *S. pseudintermedius* 16S rRNA nucleotide sequence data obtained in this study are available in GenBank using the accession numbers OQ842281-OQ842285.

Published *mecA* sequences in the GenBank database originating from other global locations were used to compare all sequences including *S. aureus* (KX68974), *S. pseudintermedius* (CP031605 and GU301100), *S. haemolyticus* (JQ764731), and *S. aureus mecC* (KX018811) for the phylogenetic tree. The *S. pseudintermedius mecA* nucleotide sequence data obtained in this study are available in GenBank using the accession numbers OQ852490-852494.

### 4.5. Antimicrobial Susceptibility Testing

Antimicrobial susceptibility tests were conducted using the Kirby–Bauer disk-diffusion method on Mueller–Hinton agar (MHA) (Clinical Diagnostics Ltd., Bangkok, Thailand) according to the Clinical and Laboratory Standards Institute (CLSI) guideline [73]. The bacterial samples, *mecA*-negative *S. pseudintermedius* (n = 23 representative of approximate 50% of *S. pseudintermedius*) and *mecA*-positive *S. pseudintermedius* (n = 10), were prepared by suspending colonies in 0.85% NaCl to an optical density (OD) 0.5 McFarland standard turbidity using a densitometer (DEN-1B, Biosan, Gibthai Co., Ltd., Bangkok, Thailand). Then, the bacterial suspension was streaked on MHA using sterile cotton swabs and incubated with the selected antimicrobial disks from several antimicrobial classes, including penicillin (oxacillin [1 µg], cloxacillin [5 µg], and ampicillin [10 µg]), beta-lactamase inhibitor (amoxicillin–clavulanic acid [30 µg]), cephalosporins (cephalexin [30 µg], cefoxitin [30 µg], ceftriaxone [30 µg], and cefotaxime [30 µg]), fluoroquinolones (norfloxacin [10 µg] and enrofloxacin [5 µg]), sulfonamide/dihydrofolate reductase inhibitor (sulfamethoxazole/trimethoprim [25 µg]), tetracyclines (doxycycline [30 µg]), aminoglycosides (amikacin [30 µg] and gentamicin [10 µg]), macrolides (erythromycin [15 µg]), and lincosamides (clindamycin [2 µg]) (Clinical Diagnostics Ltd., Bangkok, Thailand). Then, agar plates with antimicrobial disks were incubated at 35 °C ± 2 °C for 16–20 h. The inhibition zone diameter values were recorded (mm). Isolates were categorized on the basis of antimicrobial susceptibility as susceptible, intermediate, or resistant according to the breakpoint criteria recommended by the CLSI. *S. aureus* ATCC 25923 and methicillin-resistant *S. aureus* ATCC 33591 were used as the reference strains. *S. pseudintermedius* isolates showing resistance against ≥3 antimicrobial classes were defined as MDR isolates [74].

### 4.6. Determination of MIC and MBC

A single colony from each isolate, *S. pseudintermedius* (n = 23) and MRSP (n = 10), was suspended in 0.85% NaCl and adjusted turbidity to 0.5 McFarland standard. The standardized 0.5 McFarland bacterial suspension was prepared in Mueller–Hinton broth (MHB, Becton Dickinson & Co.; Sparks, MD, USA) to achieve a final concentration of approximately 1 × 10^5^ colony-forming units (CFU)/mL [75]. The plant extracts were prepared the stock solutions in MHB (100 mg/mL DMSO). The concentrations were diluted via serial two-fold dilution, with the final concentration ranging from 64 to 2048 µg/mL. Each concentration (100 µL) of plant extracts was added to 96-well plates. Subsequently, 100 µL of each bacterial inoculum was added to 96-well plates containing plant extract solution. Triplicate treatments were made for each isolate, MHB with DMSO, but no extracts were used as a growth control. To verify the sterility of the procedure, ceftriaxone was used as a positive control. The treated plates were incubated for 24 h at 37 °C. Resazurin solution (0.015%) was added to all wells (30 µL/well) and further incubated for 2 h. Viable bacteria could reduce resazurin, thereby changing its color from blue to purple or pink. Therefore, the MIC was defined as the lowest extract concentration that had no change in color (remained blue) [76].

MBC was determined by streaking 5 µL from each well with an extract concentration equal to or greater than the MIC value on MHA. The MHA plates were incubated at 37 °C for 24 h. The MBC values were considered the lowest concentrations where colonies did not grow.

### 4.7. Determination of Antibiofilm Activity

The clinical isolations of 10 *S. pseudintermedius* isolates which were systematic sampling from a total of 23 samples and 10 MRSP isolates were grown on sheep blood agar; a single colony was suspended in 0.85% NaCl, and turbidity was adjusted to the 0.5 McFarland standard suspension. Then, the bacteria were suspended in Tryptic Soy Broth (Clinical Diagnostics Ltd., Bangkok, Thailand) and adjusted to the final concentration of 1 × 10^6^ CFU/mL. A volume of 100 µL was added to each well and incubated for 24 h (irreversible attachment phase) [55].

The supernatant was discarded, and each well was gently washed three times with 200 µL of sterile phosphate-buffered saline (PBS). Residual adherent bacteria were fixed with 200 µL of 95% methanol and stained with 150 µL of 0.3% crystal violet for 15 min at RT. The excess dye was removed using PBS. The plates were air-dried, and the stained biofilms were resolubilized in 150 µL of 33% (*v*/*v*) glacial acetic acid. The OD was measured using a micro-ELISA automatic plate reader (BIOTEK, Winooski, VT, USA) at 590 nm to quantify biofilm formation. Each experiment was conducted three times [77]. The biofilm formation cutoff was established according to the OD, and OD was used for biofilm gradation. OD control = OD average of negative control + (3 × SD of OD of negative control), and OD treatment referred to the OD of the treatment wells. The biofilm formation ability was classified as [17,78] OD treatment ≤ OD control = non-biofilm producer, OD control < OD treatment ≤ 2OD control = weak biofilm producer, 2OD control < OD treatment ≤ 4OD control = moderate biofilm producer, and 4OD control ≤ OD treatment = strong biofilm producer.

### 4.8. Biofilm Detection via XTT

After assessing the biofilm-forming ability, the media and free-living bacteria were discarded. Then, 200 μL of aliquot of plant extracts was added on a 96-well plate to achieve the final concentration of MIC (512 µg/mL), 2× MIC (1024 µg/mL), 4× MIC (2048 µg/mL), and 8× MIC (4096 µg/mL), and the plates were incubated further at 37 °C for 24 h. After incubation, biofilm formation was determined via XTT [2,3-bis-(2-methoxy-4-nitro-5-sulfophenyl)-2H-tetrazolium-5-carboxanilide] assay.

The activated-XTT solution was prepared according to the manufacturer’s protocols. Briefly, 100 μL of activation reagent was added to 5.0 mL of XTT reagent (Sigma-Aldrich, St. Louis, MO, USA). To determine biofilm biomass, 50 µL of the activated-XTT solution was added to each well of the 96-well plate and incubated in the dark for 2 h and quantified using a microplate reader (BIOTEK, Winooski, VT, USA) at 490 nm [79].

### 4.9. GC–MS

The chemical composition of the PB extracts was analyzed using GC–MS. The stock samples (1 mg/mL) were diluted in methanol (1–10 µg/mL). Then, the stock samples were injected in the split mode (1:10 split ratio) into the GC–MS model Agilent 7890A/5977B GC/MSD system with 19091S-433 capillary column (0.25 µm film thickness × 0.25 mm diameter × 30 m length) (Agilent Tech., Santa Clara, CA, USA) at a flow rate of 1 mL·min^−1^ in helium as the carrier gas and an injector temperature of 250 °C. The initial oven temperature was set to 70 °C for 5 min and ramped to 250 °C at 10 °C/min, with a final hold of 5 min. The ion source temperature during MS was 230 °C, along with an ionization energy of 70 eV and a mass scan range of 35–550 m/z. The compounds were identified by matching the GC–MS results with the retention time and spectral database of the NIST library.

### 4.10. Statistical Analysis

Data were evaluated for normal distribution using the Shapiro–Wilk test prior to one-way analysis of variance. All statistical analyses were conducted using IBM SPSS Statistics version 21.0. A *p* value of <0.05 was considered statistically significant.

## 5. Conclusions

Approximately 90% of the MRSP isolated from dogs showing clinical signs of pyoderma had multidrug resistance profiles. The results of this study indicated that PB exerts antibacterial and antibiofilm effects against *S. pseudintermedius* and MRSP, with its major components being hydroxychavicol (36.02%), allylpyrocatechol diacetate (17.56%), and chavibetol (12.3%). PB is a potential candidate for the treatment of MRSP and its biofilms. The effects of PB on biofilm-associated genes and the PB-derived compounds, including the mechanism on antimicrobial activity, should be investigated. Furthermore, the development of PB formulation for canine pyoderma treatment should be studied in detail in the future.

## Figures and Tables

**Table 1:** Minimal inhibitory concentrations (MIC) of the ethanolic extracts of *Piper betle*, *P. sarmentosum*, and *P. nigrum* on bacterial isolates.

Plant Extracts	(MIC) (µg/mL)	Bacterial Isolates			
		SA	MRSA	SP	MRSP
		ATCC 25923	ATCC33591	(n = 23)	(n = 10)
PB	MIC_50_	1024	256	256	512
	MIC_90_			512	512
	GM of MIC			362.04	477.71
	MIC range			256–1024	256–1024
PN	MIC_50_	128	512	1024	2048
	MIC_90_			4096	4096
	GM of MIC			955.43	2352.53
	MIC range			128–4096	2048–4096
PS	MIC_50_	4096	1024	1024	1024
	MIC_90_			1024	2048
	GM of MIC			955.43	1351.18
	MIC range			256–4096	1024–2048

MIC_50_, concentration at which ≥50% of the isolates were inhibited; MIC_90_, concentration at which ≥90% of the isolates were inhibited; GM, geometric mean; SA, *Staphylococcus aureus* ATCC 25923; MRSA, methicillin-resistant *S. aureus* ATCC33591; SP, *Staphylococcus pseudintermedius*; MRSP, methicillin-resistant *S. pseudintermedius*; PB, *Piper betle*; PS, *P. sarmentosum*; PN, *P. nigrum*.

**Table 2:** Chemical composition of *Piper betle* ethanolic extracts.

No.	Retention Time (min)	Classes	Compounds	Formula	Chemical Structure	Peak Area (%)
1	6.82	Alkanes	Undecane	C_11_H_24_	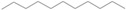	0.17
2	8.3	Phenols	Catechol	C_6_H_6_O_2_	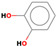	0.377
3	8.55	Coumarins	Benzofuran, 2,3-dihydro-	C_8_H_8_O	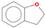	0.3
4	9.03	Propenylphenols	Chavicol	C_9_H_10_O	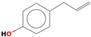	0.174
5	10.65	Propenylphenols	Chavibetol	C_10_H_12_O_2_	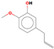	12.3
6	11.38	Sesquiterpene	Caryophyllene	C_15_H_24_	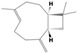	0.857
7	11.9	Phenols	Hydroxychavicol	C_9_H_10_O_2_	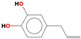	36.02
8	12.03	Sesquiterpene	γ-Muurolene	C_15_H_24_	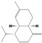	2.05
9	12.13	Sesquiterpene	Germacrene D	C_15_H_24_	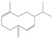	0.77
10	12.32	Benzoic acid	3,5-Dimethylbenzoic acid	C_9_H_10_O_2_	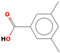	5.87
11	12.54	Phenols	Isoeugenol	C_10_H_12_O_2_	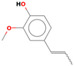	6.78
12	13.9	Propenylphenols	Allylpyrocatechol diacetate	C_13_H_14_O_4_	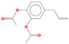	17.56
13	16.71	Alkaloids	Piperidine	C_5_H_11_N	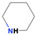	3.17
14	17.44	Benzodioxoles	Piperlonguminine	C_16_H_19_NO_3_	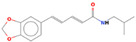	0.04
15	18.49	Diterpene	Phytol	C_20_H_40_O	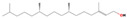	1.28
16	18.58	Fatty acid methyl esters	Methyl stearate	C_19_H_38_O_2_	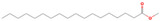	0.15
17	19.39	Sesquiterpene	Neophytadiene	C_20_H_38_	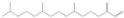	0.4
18	19.93	Alkaloids	Piperine	C_17_H_19_NO_3_	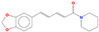	0.7
Total						88.97

**Table 3:** Thermocycler conditions for amplification of *nuc*, *mecA*, and 16S rRNA.

Stages	Gene Target		
	*nuc*	*mecA*	16S rRNA
Stage 1: 1 cycle	3 min step at 94 °C	3 min step at 94 °C	3 min step at 94 °C
Stage 2: 30 cycles	30 s at 94 °C	30 s at 94 °C	30 s at 94 °C
	30 s at 50 °C	30 s at 58 °C	30 s at 55 °C
	60 s at 72 °C	60 s at 72 °C	60 s at 72 °C
Stage 3: 1 cycle	5 min at 72 °C	5 min at 72 °C	5 min at 72 °C

**Table 4:** Oligonucleotide primers for polymerase chain reaction of *Staphylococcus pseudintermedius* and *mecA*.

Species	Primer	Sequence (5′-3′)	Gene Target	Expected Size (bp)	References
*S. pseudintermedius*	pse-F2 pse-R5	5′-TRGGCAGTAGGATTCGTTAA-3′ 5′-CTTTTGGTGCTYCMTTTTGG-3′	*nuc*	926	[64]
*Staphylococcus* spp.	MecA1 MecA2	5′-CCAATTCCACATTGTTTCGGTCTAA-3 5′-CCAATTCCACATTGTTTCGGTCTAA-3′	*mecA*	310	[69]
*Staphylococcus* spp.	Staph 756-F Staph 750-R	5′-AACTCTGTTATTAGGGAAGAACA-3′ 5′-CCACCTTCCTCCGGTTTGTCACC-3′	16S rRNA	756	[70]

## Data Availability

The data presented in this study are available within the article.

## Supplementary Materials

The following supporting information can be downloaded at https://www.mdpi.com/article/10.3390/ph16050741/s1, Figure S1: PCR amplification of the *mecA* and *nuc* genes of *Staphylococcus pseudintermedius* on 2% Agarose gel electrophoresis; Figure S2: PCR amplification of the 16S rRNA gene of *Staphylococcus* spp.; Figure S3: Phylogenetic tree analysis of *Staphylococcus pseudintermedius* based on nucleotide sequences from a 701 bp fragment of 16S rRNA using the neighbor-joining method.; Figure S4: Phylogenetic tree analysis of *Staphylococcus* spp. carrying *mecA* gene based on nucleotide sequences from a 254 bp fragment of *mecA* using the neighbor-joining method.; Figure S5: Microscopic appearance of *Staphylococcus pseudintermedius* classified as weak (a), moderate (b), and strong (c) biofilm producers after a 24 h incubation (20× magnification); Figure S6: Biofilm formation values (OD590) of *Staphylococcus aureus* ATCC 25923 (SA); methicillin-resistant *S. aureus* ATCC 33591 (MRSA), methicillin-resistant *Staphylococcus pseudintermedius* (MRSP, n = 10) and *S. pseudintermedius* isolates (n = 23) obtained by crystal violet assay; Table S1: Antimicrobial susceptibility profiles of *Staphylococcus aureus*, methicillin-resistant *S. aureus*, methicillin-resistant *Staphylococcus pseudintermedius*, and *S. pseudintermedius* determined using the disk-diffusion method; Table S2: Effects of *Piper nigrum* (PN) and *P. sarmentosum* (PS) ethanolic extracts on the biofilm formation of *Staphylococcus aureus* (SA), methicillin-resistant *S. aureus* (MRSA), and *Staphylococcus pseudintermedius* after 24 h of treatment.
