# Supplementary material for: Effects of Piper betle Extracts against Biofilm Formation by Methicillin-Resistant Staphylococcus pseudintermedius Isolated from Dogs
Source: Pharmaceuticals (Basel). 2023 May 12;16(5):741. doi: 10.3390/ph16050741 (PMC10224074; doi:10.3390/ph16050741)
Supplement: Supplementary file 1 [file pharmaceuticals-16-00741-s001.zip › Supplementary Figure S1.pdf]

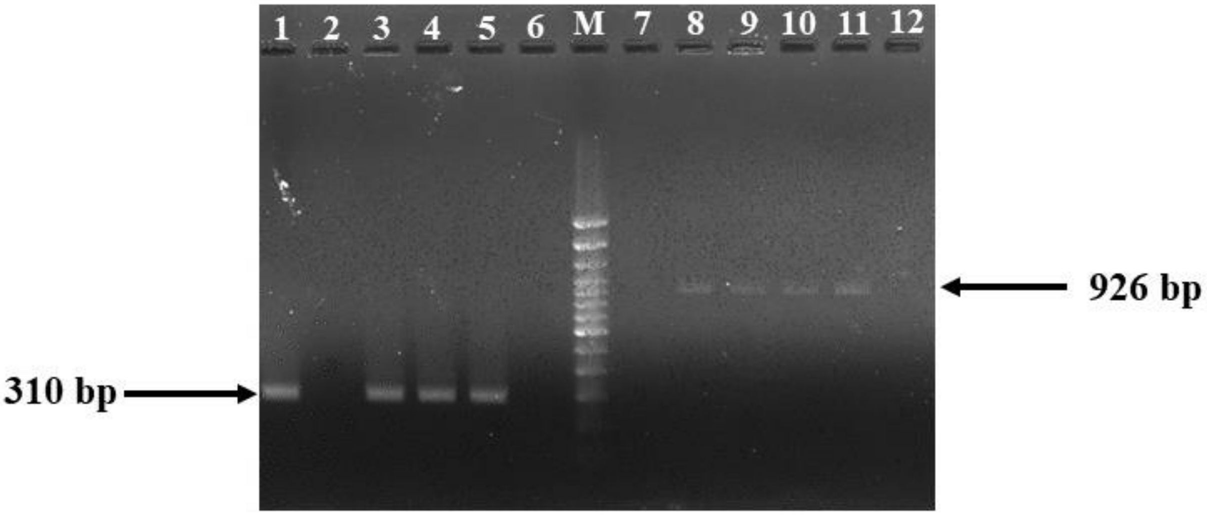

Figure S1. PCR amplification of the *mecA* and *nuc* genes of *Staphylococcus pseudintermedius* on 2% Agarose gel electrophoresis. Lane M DNA ladder, MW 100 bp ladder. Lanes 1 and 7 *S. aureus* carrying *mecA* gene showed a typical band size of 310 bp corresponding to *mecA* and negative for the *nuc* gene of *S. pseudintermedius*, respectively. Lanes 2-4 and 8-10, *S. pseudintermedius* samples. Lanes 5 and 11, positive control sample, showed a typical band size of 310 bp corresponding to *mecA* and 926 bp for *nuc* gene, respectively. Lanes 6 and 12 were the negative control (nuclease-free water).
