# Supplementary material for: Effects of Piper betle Extracts against Biofilm Formation by Methicillin-Resistant Staphylococcus pseudintermedius Isolated from Dogs
Source: Pharmaceuticals (Basel). 2023 May 12;16(5):741. doi: 10.3390/ph16050741 (PMC10224074; doi:10.3390/ph16050741)
Supplement: Supplementary file 1 [file pharmaceuticals-16-00741-s001.zip › Supplementary Figure S2.pdf]

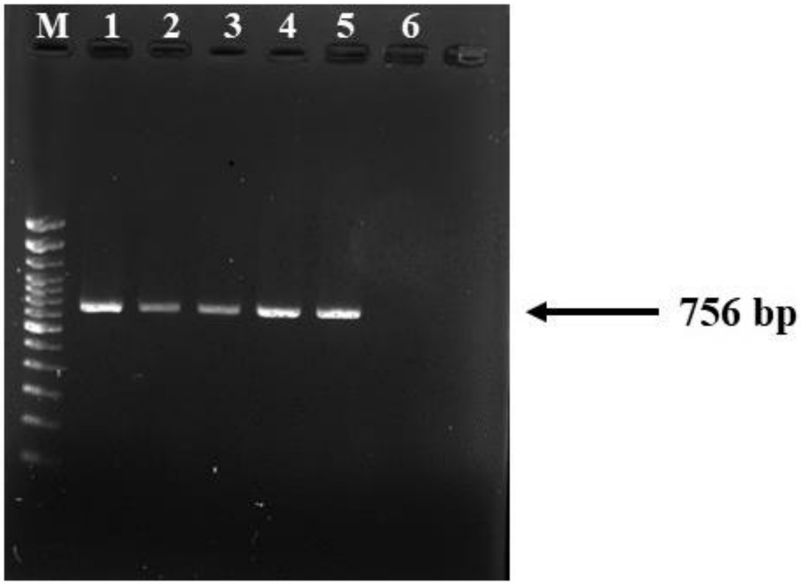

Figure S2. PCR amplification of the 16S rRNA gene of *Staphylococcus* spp. on 2% Agarose gel electrophoresis. Lane M DNA ladder, MW 100 bp ladder. Lane 1 *Staphylococcus aureus* sample, Lanes 2-5 *Staphylococcus pseudintermedius* samples, showed a typical band size of 756 bp corresponding to *16S rRNA* of the selected isolates using 16S rRNA primers, and Lane 6 was the negative control (nuclease-free water).
