# Supplementary material for: Effects of Piper betle Extracts against Biofilm Formation by Methicillin-Resistant Staphylococcus pseudintermedius Isolated from Dogs
Source: Pharmaceuticals (Basel). 2023 May 12;16(5):741. doi: 10.3390/ph16050741 (PMC10224074; doi:10.3390/ph16050741)
Supplement: Supplementary file 1 [file pharmaceuticals-16-00741-s001.zip › Supplementary Figure S3.pdf]

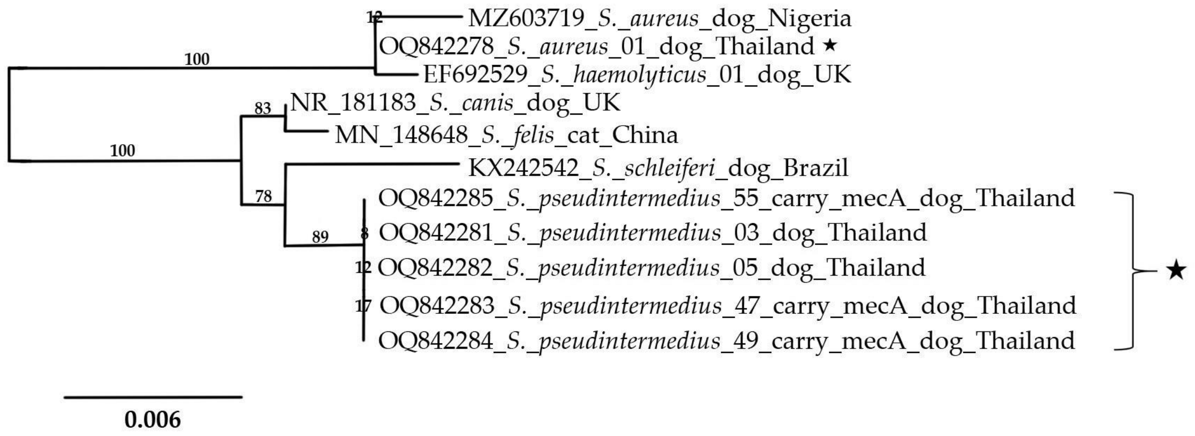

Figure S3: Phylogenetic tree analysis of *Staphylococcus pseudintermedius* based on nucleotide sequences from a 701 base pair fragment of 16S rRNA using the neighbor-joining method. Sequences from this study are marked with stars (accession numbers OQ842281-OQ842285).
