# Supplementary material for: Effects of Piper betle Extracts against Biofilm Formation by Methicillin-Resistant Staphylococcus pseudintermedius Isolated from Dogs
Source: Pharmaceuticals (Basel). 2023 May 12;16(5):741. doi: 10.3390/ph16050741 (PMC10224074; doi:10.3390/ph16050741)
Supplement: Supplementary file 1 [file pharmaceuticals-16-00741-s001.zip › Supplementary Figure S4.pdf]

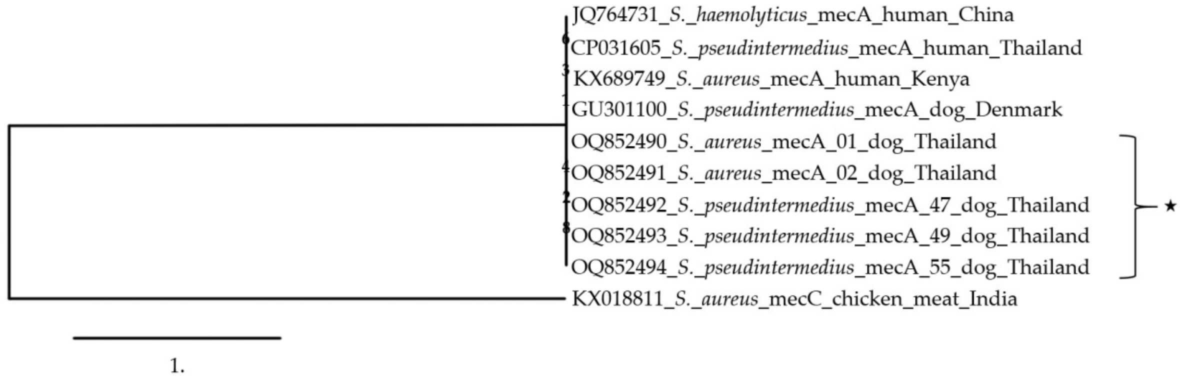


**Figure S4:** Phylogenetic tree analysis of *Staphylococcus* spp. carrying *mecA* gene based on nucleotide sequences from a 254 base pair fragment of *mecA* using the neighbor-joining method. Sequences from this study are marked with stars (accession numbers OQ852490-852494).
