# Supplementary material for: Effects of Piper betle Extracts against Biofilm Formation by Methicillin-Resistant Staphylococcus pseudintermedius Isolated from Dogs
Source: Pharmaceuticals (Basel). 2023 May 12;16(5):741. doi: 10.3390/ph16050741 (PMC10224074; doi:10.3390/ph16050741)
Supplement: Supplementary file 1 [file pharmaceuticals-16-00741-s001.zip › Supplementary Figure S5.pdf]

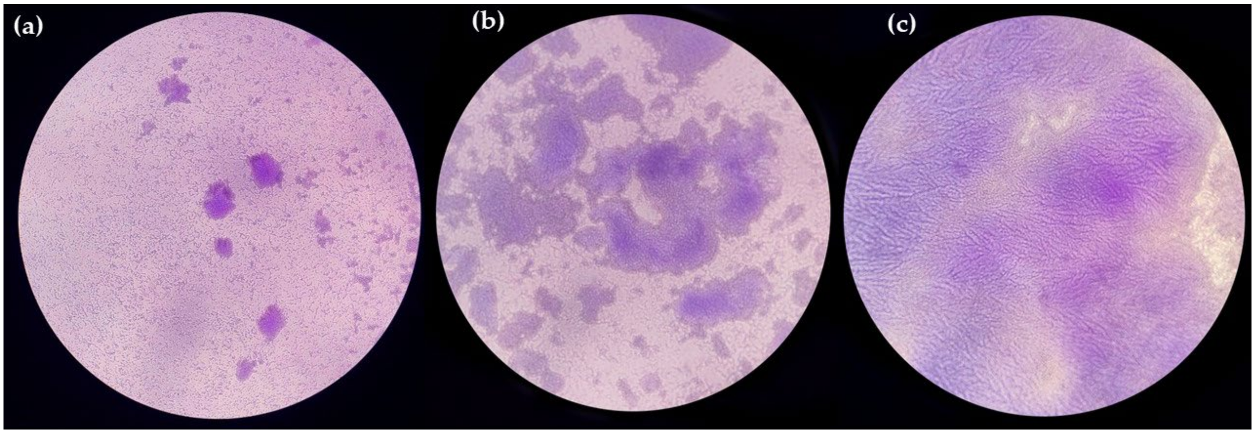

Figure S5. Microscopic appearance of *Staphylococcus pseudintermedius* classified as weak (a), moderate (b), and strong (c) biofilm producers after a 24h incubation (20× magnification).
