# Supplementary material for: Effects of Piper betle Extracts against Biofilm Formation by Methicillin-Resistant Staphylococcus pseudintermedius Isolated from Dogs
Source: Pharmaceuticals (Basel). 2023 May 12;16(5):741. doi: 10.3390/ph16050741 (PMC10224074; doi:10.3390/ph16050741)
Supplement: Supplementary file 1 [file pharmaceuticals-16-00741-s001.zip › Supplementary Figure S6.pdf]

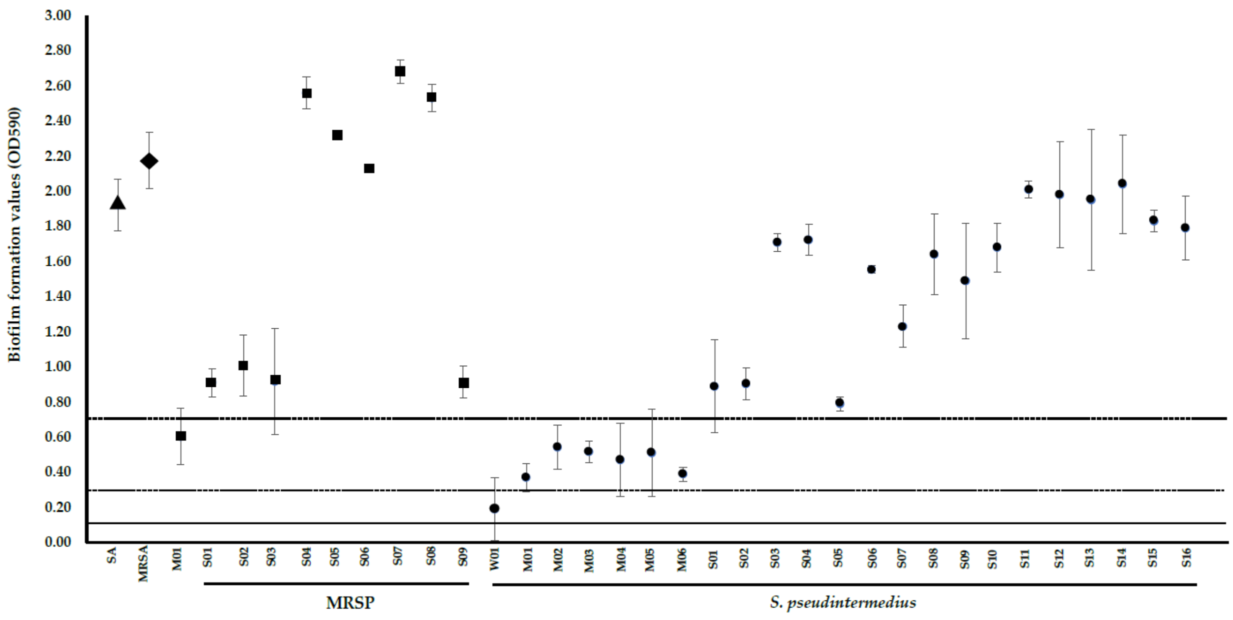

Figure S6. Biofilm formation values (OD590) of *Staphylococcus aureus* ATCC 25923 (SA); methicillin-resistant *S. aureus* ATCC 33591 (MRSA), methicillin-resistant *Staphylococcus pseudintermedius* (MRSP, n=10) and *S. pseudintermedius* isolates (n=23) obtained by crystal violet assay. The OD cut-off used to distinguish weak and moderate biofilm producers from strong biofilm producers is 0.18 (dashed line). Categories: non-biofilm producers (OD ≤ 0.18), weak biofilm producers (0.18 < OD ≤ 0.35 (dashed line)), moderate biofilm producers (0.35 < OD ≤ 0.70 (dashed line)), and strong biofilm producers (0.70 < OD). (▲) SA (◆) MRSA; (■) MRSP; (●) *S. pseudintermedius*).
