# Supplementary material for: Effects of Piper betle Extracts against Biofilm Formation by Methicillin-Resistant Staphylococcus pseudintermedius Isolated from Dogs
Source: Pharmaceuticals (Basel). 2023 May 12;16(5):741. doi: 10.3390/ph16050741 (PMC10224074; doi:10.3390/ph16050741)
Supplement: Supplementary file 1 [file pharmaceuticals-16-00741-s001.zip › Supplementary Table S1.pdf]

Table S1. Antimicrobial susceptibility profiles of *Staphylococcus aureus*, methicillin-resistant *S. aureus*, methicillin-resistant *Staphylococcus pseudintermedius*, and *S. pseudintermedius* determined using the disk-diffusion method.

Bacterial isolate		Antimicrobial resistance profile
SA		NOR
MRSA		OX-OB-AMP-AMC-CL-FOX-CRO-CTX-FEP-NOR-EDA
MRSP	M01	AMP-STX
	S01	AMP-CTX-SXT-DO-AK-CN-E
	S02	OX-AMP-AMC-FEP-SXT-AK
	S03	OX-OB-AMP-AMC-CL-FOX-CRO-CTX-FEP-NOR-ENR-STX-DO-AK-CN-E-DA
	S04	OX-OB-AMP-AMC-FOX-CRO-CTX-FEP-NOR-ENR-STX-DO-E-DA
	S05	OX-OB-AMP-AMC-CTX-FEP-NOR-ENR-STX-DO-CN-E-DA
	S06	OX-OB-AMP-AMC-CL-CRO-CTX-FEP-NOR-ENR-STX-DO-AK-CN
	S07	OX-AMP-CRO-CTX-FEP-NOR-ENR-DO-CN-E
	S08	OX-OB-AMP-AMC-CL-FOX-CRO-CTX-FEP-NOR-ENR-STX-DO-CN-DA
	S09	OX-AMP-CRO-CTX-FEP-NOR-ENR-E-DA
S. pseudintermedius	M01	NOR
	M02	OB-AMP
	M03	AMP-AMC
	M04	AMP-STX-DO-CN
	M05	AMP
	M06	AMP
	S01	AMP
	S02	AMP-AMC-NOR
	S03	OB-AMP-DA
	S04	OB-AMP-DA
	S05	OB-AMP-AK
	S06	OB-AMP-AMC-AK
	S07	AMP-DO
	S08	AMP-DO
	S09	AMP
	S10	AMP
	S11	AMP
	S12	AMP
	S13	AMP
	S14	AMP-DO
	S15	AMP
	S16	OB-AMP-AMC

Abbreviations: SA, *Staphylococcus aureus* ATCC 25923; MRSA, methicillin-resistant *S. aureus* ATCC 33591; W, weak biofilm producer; M, moderate biofilm producer; S, strong biofilm producer; OX, oxacillin (1 μg); OB, cloxacillin; AMP, ampicillin (10 μg); AMC, amoxicillin–clavulanic acid (30 μg); CL, cephalexin (30 μg); FOX, cefoxitin (30 μg); CRO, ceftriaxone (30 μg); CTX, cefotaxime (30 μg); NOR, norfloxacin (10 μg); ENR, enrofloxacin (5 μg); SXT, sulfamethoxazole/trimethoprim (25 μg); DO, doxycycline (30 μg); AK, amikacin (30 μg); CN, gentamicin (10 μg); E, erythromycin (15 μg); DA, clindamycin (2 μg)
