# Supplementary material for: Effects of Piper betle Extracts against Biofilm Formation by Methicillin-Resistant Staphylococcus pseudintermedius Isolated from Dogs
Source: Pharmaceuticals (Basel). 2023 May 12;16(5):741. doi: 10.3390/ph16050741 (PMC10224074; doi:10.3390/ph16050741)
Supplement: Supplementary file 1 [file pharmaceuticals-16-00741-s001.zip › Supplementary Table S2.pdf]

Table S2. Effects of *Piper nigrum* (PN) and *P. sarmentosum* (PS) ethanolic extracts on the biofilm formation of *Staphylococcus aureus* (SA), methicillin-resistant *S. aureus* (MRSA), and *Staphylococcus pseudintermedius* after 24 h of treatment.

Bacterial isolates		Percentage of biofilm inhibition			
		PN		PS	
		8 x MIC	4 x MIC	8 x MIC	4 x MIC
SA		49.89 ± 0.47	36.4 ± 0.57	N.I.	N.I.
MRSA		21.94 ± 0.39	18.75 ± 2.24	N.I.	N.I.
S. psuedintermedius	W01	23.31 ± 3.38	N.I.	N.I.	N.I.
	M02	29.38 ± 10.4	11.33 ± 5.14	17.23 ± 9.65	18.90 ± 9.25
	M04	N.I.	N.I.	N.I.	N.I.
	M06	N.I.	N.I.	N.I.	N.I.
	S02	N.I.	N.I.	N.I.	N.I.
	S04	16.45 ± 1.7	13.75 ± 6.75	12.51 ± 6.08	7.84 ± 3.79
	S06	7.72 ± 2.45	N.I.	N.I.	N.I.
	S08	N.I.	N.I.	3.43 ± 0.12	N.I.
	S10	N.I.	N.I.	N.I.	N.I.
	S12	N.I.	N.I.	N.I.	N.I.
MRSP	M01	10.39 ± 5.58	N.I.	N.I.	N.I.
	S01	N.I.	N.I.	N.I.	N.I.
	S02	N.I.	N.I.	N.I.	N.I.
	S03	N.I.	N.I.	N.I.	N.I.
	S04	14.42 ± 40.3	5.82 ± 2.01	N.I.	N.I.
	S05	18.89 ± 1.56	17.14 ± 4.9	3.05 ± 1.55	N.I.
	S06	N.I.	N.I.	N.I.	N.I.
	S07	N.I.	N.I.	N.I.	N.I.
	S08	N.I.	N.I.	N.I.	N.I.
	S09	N.I.	N.I.	N.I.	N.I.

Abbreviations: SA, *Staphylococcus aureus* ATCC 25923; MRSA, methicillin-resistant *S. aureus* ATCC 160 33591; MRSP, methicillin-resistant *S. pseudintermedius*; N.I., non-inhibition; W, weak; M, moderate; and S, strong biofilm producers.
